# Reduction of Arterial Stiffness Assessed by Cardio-Ankle Vascular Index Through Intra-Dialytic Aerobic Training

**DOI:** 10.3390/jcm15208005

**Published:** 2026-10-15

**Authors:** Alena Havelková, Petr Dobšák, Petr Dvořák, Michal Pohanka, Alena Pokorná, Marie Nováková, Petr Filipenský

**Affiliations:** 1 Department of Sports Medicine and Rehabilitation, St. Anne’s University Hospital in Brno, Faculty of Medicine, Masaryk University, 602 00 Brno, Czech Republic; petr.dobsak@fnusa.cz (P.D.); alena.pokorna@fnusa.cz (A.P.); 2 Department of Physical Activities and Health Sciences, Faculty of Sports Studies, Masaryk University, 625 00 Brno, Czech Republic; 3 Department of Biology and Wildlife Diseases, Faculty of Veterinary Hygiene and Ecology, University of Veterinary Sciences, 612 42 Brno, Czech Republic; dvorakp@vfu.cz; 4 Department of Physiology, Faculty of Medicine, Masaryk University, 625 00 Brno, Czech Republic; 5 Department of Urology, St. Anne’s University Hospital in Brno, Faculty of Medicine, Masaryk University, 602 00 Brno, Czech Republic

**Keywords:** arterial stiffness, cardio-ankle vascular index, hemodialysis, intradialytic exercise, functional capacity, cardioprotective effect

## Abstract

**Background**: Increased arterial stiffness elevates mortality in chronic kidney disease. Limited available data suggest that regular exercise can reduce arterial stiffness in hemodialysis patients. This pilot study aimed to investigate the effects of intra-dialytic aerobic training on arterial stiffness in hemodialysis patients. **Methods**: Outpatients undergoing hemodialysis were randomized to a 20-week intra-dialytic aerobic training group (bedside ergometer exercise) or a control group receiving standard care. Arterial stiffness was evaluated using the cardio-ankle vascular index (CAVI). CAVI measurements, functional fitness tests, laboratory analyses, and quality-of-life assessments were performed at baseline and at the end of the study. **Results**: A significant decrease in CAVI was observed in the intra-dialytic aerobic training group (−5%; *p* = 0.037). Concurrently, significant improvements occurred in VO2peak/kg (+31%; *p* = 0.015), 6 min walk test (6 MWT) distance (+18%; *p* = 0.005), clearance (URR +2%; *p* < 0.001; Kt/V +6%; *p* = 0.001), and select quality-of-life parameters (social support +9%; *p* = 0.022; general health +25%; *p* = 0.008; well-being +3%; *p* < 0.001). No significant changes were found in the control group. **Conclusions**: The reduction in arterial stiffness confirms the efficacy of intra-dialytic aerobic training in improving overall health status. Consequently, CAVI appears to be a valuable tool for monitoring arterial stiffness changes in hemodialysis patients.

## 1. Introduction

Technological progress and a number of breakthrough discoveries in the pharmaceutical industry have led to the successful treatment of previously hopeless fatal diseases in recent decades. At the same time, however, the incidence of so-called age-dependent chronic pathologies, including chronic kidney disease (CKD), is increasing [1]. The irrepressible loss of excretory and regulatory functions culminates in the development of uremic syndrome and CKD, which requires urgent intervention with substitution therapy, such as dialysis or transplantation. Patients at various stages of CKD suffer from a number of concomitant comorbidities, especially cardiovascular (CV), which are considered the leading cause of disease progression and extremely high mortality [2,3,4]. Chronic kidney disease confers an elevated cardiovascular risk that extends beyond the traditional risk factors of hypertension and diabetes, thereby establishing cardiovascular disease as a critical target for therapeutic interventions. Given that cardiovascular disease represents the primary cause of mortality among individuals with kidney failure undergoing hemodialysis, enhancing cardiovascular health remains a paramount clinical priority for this population [5]. However, clinical trials of interventions targeting atherosclerosis, such as statin therapy, have yielded disappointing results in patients undergoing dialysis. This lack of efficacy may be attributed to a higher prevalence of heart failure and sudden cardiac death relative to that observed among individuals with traditional atherosclerotic coronary disease. Particularly in hemodialysis patients, disrupted cardiovascular physiology during the procedure induces intradialytic hypotension (IDH) and recurrent cardiac ischemia, subsequently driving wall motion abnormalities and left ventricular dysfunction, a phenomenon collectively conceptualized as myocardial stunning [6].

Increased arterial stiffness (AS) is closely related to the rise of CV mortality, which develops much more aggressively in patients with CKD than in healthy individuals or other chronic pathologies [7,8]. Importantly, arterial stiffness (AS), which serves as a surrogate marker for endothelial dysfunction, has been established as a robust predictor of cardiovascular mortality in patients with chronic hemodialysis [9]. Extremely dangerous pathological potential is represented in particular by permanent exposure to uremic toxins (e.g., reactive substances, advanced glycation end-products, carbamylation products, etc.), high plasma concentration of pro-inflammatory substances (e.g., IL-6, TNF-alpha, CRP, etc.), limited availability of nitric oxide [10,11,12], and also certain negative side effects of the dialysis procedure itself [13,14]. The resulting state of permanent pro-inflammatory environment severely disrupts the structural-functional properties of arterial walls, total pathological remodeling of the arterial system, and progression of CV disease [10,11,12]. Thus, the close connection between the growth of AS and the progression of CV pathologies in hemodialysis (HD) patients should be sufficient motivation for regular monitoring of AS in dialysis patients. This has unquestionable clinical significance not only for assessing the state of arterial changes, but also for evaluating the effectiveness of therapeutic interventions and prognosis [15].

The positive health benefits of regular physical activity have been repeatedly demonstrated in a number of chronic diseases [16,17]. The human body quickly adapts to an inactive lifestyle, and if no correction is made within a certain time horizon, the progression of chronic pathologies is unhindered, resulting in both reduced quality of life (QoL) and high mortality [18]. This fact further emphasizes the need for and importance of regular physical activity as part of a routine treatment strategy for dialysis patients. It has been known for several decades that exercise training and regular physical activity in dialysis patients have a number of extraordinary positive effects, including overall significant stabilization of health status, reduction of CV risk, and QoL improvement [19,20]. Various methods of intra-dialytic or extra-dialytic (days without dialysis) training of aerobic, resistance, or combined types have been applied with varying success [21,22,23,24]. However, the effect of physical activity on AS in HD patients has been investigated to date by only a limited number of clinical studies. Their conclusions show that exercise training of various types and durations can significantly reduce AS [25,26,27] and thus reduce CV risk.

All the aforementioned studies used traditional and standard methodologies to measure AS, i.e., pulse wave velocity (PWV) or augmentation index (Aix) [9,25,26,27]. However, in the current literature there is no information on the evaluation of the effects of intradialytic training (IDT) on AS using a newer and more accurate measurement method, based on the parameter CAVI [28]. Thus, the primary objective of this randomized trial was to assess the effect of 20 weeks of IDT on AS as measured by CAVI in a group of HD patients. The secondary objectives focused on the evaluation of the effect of IDT on selected functional and laboratory parameters and QoL.

## 2. Methods

### 2.1. Patients and Methodology

The study was conducted in the Dialysis Centre of the 2nd Department of Internal Medicine, St. Ann’s University Hospital (Brno, Czech Republic, EU) using Fresenius 4008 S hemodialysis units (Fresenius Medical Care AG, Bad Homburg vor der Höhe, DE)Bad Homburg, Germany) and Fresenius F70S or Fresenius F8HPS S capillary dialyzers (Fresenius Medical Care AG, Bad Homburg vor der Höhe, DE). All patients signed an Informed Consent in accordance with confidentiality and applicable data protection regulations. Patients enrolled in the study underwent standard HD 3 times a week.

Inclusion criteria: age ≥ 18, HD for at least 3 months (Kt/V ≥ 1.2), symptomatic stability, and optimized unchanged pharmacological treatment a month before the start of the study.

Exclusion criteria: age < 18 years, recent ischemic cardiovascular disease, uncompensated hypertension, uncompensated diabetes mellitus, chronic heart failure (NYHA III and IV), vascular access in the lower extremities, and generally poor health status due to other causes.

The primary outcome was to assess the effect of ambulatory supervised IDT on AS, assessed by CAVI.

The secondary outcomes were to assess the effect of IDT on functional parameters (VO_2peak_/kg, VO_2_VT-1/kg, and distance walked in 6 min), on plasma concentrations of selected biochemical parameters, and on quality of life using the Kidney Disease Quality of Life-36 (KDQoL-36) questionnaire.

Given the pilot nature of this study and the fact that multiple secondary outcomes were evaluated without statistical correction for multiple comparisons, these findings are considered exploratory and hypothesis-generating. Consequently, they should be interpreted with caution and require validation in future confirmatory trials.

AS measurement. CAVI was measured by the VaSera^®^ 1500 vascular screening system (Fukuda Denshi Co., Ltd., Tokyo, Japan) following a standard protocol described in detail previously [28]. In brief, the examination was performed in the supine position, with 4 pressure cuffs placed on all limbs, a phonocardiogram above the upper edge of the sternum, and 2 ECG leads on both upper limbs. The device software automatically calculated CAVI. Patients with an ankle-brachial index (ABI) < 0.9 were excluded from the evaluation.

### 2.2. Functional Fitness Testing

Spiroergometry test. Before starting the IDT program, all patients underwent a graded bicycle spiroergometry (CPX) according to a standardized protocol [26], with 12-lead electrocardiography (AT-104 PC, Schiller^®^, Baar, Switzerland) to determine peak aerometabolic capacity (VO_2peak_/kg) and the first ventilatory threshold VT-1 (VO_2_VT-1/kg). The test was performed on a calibrated, electrically braked cycle ergometer (Ergoselect, Ergoline^®^, Bitz, Germany), with workload increments of 10 Watts. Minute ventilation and blood gas exchange were measured breath-by-breath using an automated measurement system (Power Cube, Ganshorn^®^ Medizin Electronic, Niederlauer, Germany). Blood pressure (BP) was monitored manually. Training heart rate (HR) for patients in the IDT group was determined based on the first ventilation threshold (VT-1) by the V-slope method and analysis of ventilation equivalents [29]. The VO_2peak_ value was expressed as the highest value of O_2_ consumption (O_2_ uptake) during the last 30 s of the CPX.

The 6 min walk test (6 MWT). The 6 MWT is a standard and validated diagnostic tool for assessing physical performance at submaximal intensity. At baseline and after 20 weeks, all patients completed the 6 MWT on a precisely defined 30 m track with two plastic cones indicating the turning point. During the test, HR was continuously monitored using the SF-Tester Polar S725-X (Polar Electro^®^ Oy, Kempele, Finland). Subjective fatigue and dyspnea according to the modified Borg scale (6–20) were registered at 1 min intervals [30]. The test was performed in accordance with the current American College of Sports medicine (ACSM) guidelines and recommendations and under expert supervision [31]. All functional tests were always performed on non-dialysis days in the period 1 week before the start and 1 week after the end of the study. Measurements were not repeated. BP measurement was done always in a sitting position and after 30 min of rest. Patients were asked not to consume caffeine, alcohol or exercise 24 h prior to testing.

Laboratory. Before and after 20 weeks of IDT, plasma concentrations of the following biochemical parameters were assessed in both groups: hemoglobin (Hb), albumin, glucose, calcium, phosphorus, potassium, parathyroid hormone, total cholesterol and triglycerides (TAG), creatinine, CRP, urea, urea reduction ratio (URR) and Kt/V.

Quality-of-life questionnaire. KDQoL-36 (Czech version) questionnaire was chosen to assess the health-related QoL. This questionnaire is a frequently used and certified QoL assessment tool in patients with chronic renal insufficiency due to its validity and accuracy [32].

Training protocol. Patients in the IDT group completed a 20-week exercise-based renal rehabilitation program consisting of exercises on special programmable bicycle ergometers (MOTOmed^®^ letto-2, Reck-Technik GmbH & Co, Betzenweiler, Germany) 3 times a week in supine or semi-supine position. HR was monitored continuously by an ear clip. Intra-dialytic training was always performed between the 2nd and the 3rd hour of HD. The standard IDT protocol was as follows: before connecting to the dialyzer, patients performed a series of stretching (5 min) and strengthening (5 min) exercises of the muscles of the upper and lower extremities (using dumbbells and elastic bands). This was followed by the aerobic phase: (a) passive (motor-supported) pedaling on the ergometer (1 min forward + 1 min backward); (b) active training on the ergometer (2 × 20 min with a 5 min break, i.e., passive pedaling); and (c) end-phase of passive pedaling (1 min forward + 1 min backward). The intensity of the workload was gradually increased, so that the target HR (90% of the VT-1 level) was reached during the first 10 min. Then, the patients pedaled at this intensity for the remaining training period. After disconnection from the dialyzer, another series of stretching (5 min) and strengthening (5 min) exercises were performed again. The total duration of one training unit was 60 min. During the active (aerobic) phase of the IDT, the patients were regularly asked about dyspnea, discomfort or fatigue using the Borg scale. Professional staff (doctor, physiotherapists and nurses) were present throughout the IDT.

Notice: a significant loss of muscle mass affects especially the lower extremities of HD patients, which tends to be a major handicap for performing the workload on the ergometer. Therefore, in accordance with the functional state of the patients and the “preconditioning” of the loaded muscles, during the first 2–3 weeks of IDT the active phase was shortened to 20–30 min, with an intensity below the determined VT-1 level (approx. 50–70% of HR at VT-1 according to individual tolerance). This training protocol modification allowed all the subjects to gradually adapt to and tolerate the determined workload intensity (at VT-1) in the further course of the IDT rehabilitation program.

### 2.3. Statistics

All data analyses were performed using R version 4.2.3 (R: A language and environment for statistical computing. R Foundation for Statistical Computing, Vienna, Austria). The significance level was set at *p* < 0.05. All measurements (change from baseline to 20 weeks value) were compared between the control group and the IDT group using a linear regression model adjusting for age, sex, and hypertension status. Regression coefficients describing the difference in means (95% CI) between the groups with respect to the adjusting variables are reported. Spearman’s correlation coefficient was used to calculate the correlation.

## 3. Results

**Figure 1:**
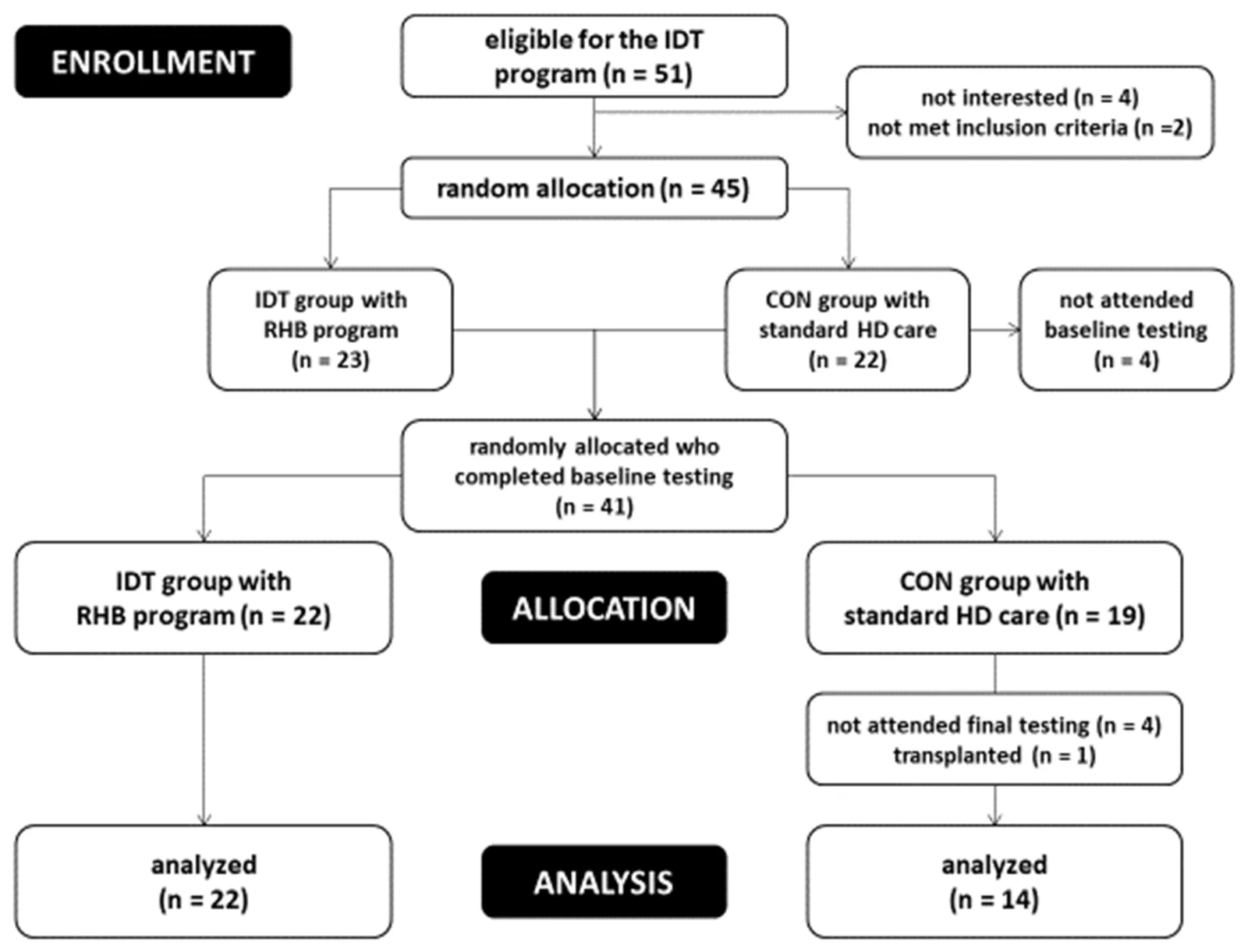
Flowchart of patients enrolled into the trial.

This randomized pilot study was conducted in a single-center outpatient hemodialysis center at St. Anne’s University Hospital Brno (Czechia). Initially, 51 patients were approached, from which a total of 45 were selected, and these were subsequently randomized by an independent statistician (not a member of the research team) using computer-generated random number software into two groups (CONSORT diagram—Figure 1). Allocation concealment and blinding of outcome assessors was maintained.

Statistical analyses followed a per-protocol approach. Participants who did not complete the final testing or underwent transplantation were excluded from the final analysis. Missing data were handled using a complete-case analysis approach. Due to the small sample size and the specific nature of the missingness, data imputation methods were deemed unfeasible and statistically unsuitable. Consequently, only participants with complete datasets across all time points were included in the final evaluation.

The final evaluation included 22 patients in the IDT group who completed a 20-week intradialytic aerobic training on bicycle ergometers. The average adherence (% of training) in the IDT group (*n* = 22) was 76% (19). The control group (*n* = 14) consisted of patients who were provided with the standard dialysis procedure without any additional intervention during the study. The mean age was 54.68 (15.24) years in the IDT group and 56.64 (16.11) years in the controls. Women accounted for 12% of all patients. Most baseline values, including the distribution of comorbidities, were comparable in both groups. Covariates for multivariable adjustment were selected based on clinical relevance. Specifically, sex, diabetes prevalence, and baseline blood pressure were included as covariates in the adjusted models to control for potential confounding effects on arterial stiffness measurements. Detailed baseline characteristics of patients in both groups are summarized in Table 1.

Primary outcome. The results of the CAVI parameter evaluation indicate that the 20-week ID aerobic exercise program led to a significant decrease in AS in the IDT group (−0.45, i.e., a decrease of 5% compared to baseline; *p* = 0.037). In contrast, the mean CAVI value in the control group did not change significantly (Table 2).

Secondary outcomes are considered exploratory. The IDT significantly improved peak oxygen uptake (VO_2peak_) (+31%; *p* = 0.015) and oxygen uptake at VT-1 (+23%; *p* < 0.001). Similarly, the distance covered in the 6 MWT increased in the IDT group (+18%; *p* = 0.005). A significant decrease in inter-dialytic DBP was found in the IDT group (−9%; *p* = 0.01), while SBP changes were not significant (−5%; NS). There were no significant changes in inter-dialytic body weight in either group. The results of the functional fitness tests are presented in Table 3, Table 4 and Table 5.

The evaluation of the KDQoL-36 questionnaires revealed that 20 weeks of IDT improved the global score of most QoL items in the exercise group (Table 6), but only three of them reached statistical significance: social support (+9%; *p* = 0.022), general health (+25%; *p* = 0.008), and well-being (+3%; *p* < 0.001).

A positive effect of IDT was observed in the results of selected laboratory parameters. Compared to the initial values, the plasma CRP level significantly decreased (−22%; *p* = 0.025) and urea reduction ratio (URR) (+2%; *p* < 0.001) and Kt/V (+6%; *p* = 0.001) significantly improved in the IDT group. Only insignificant changes were found in creatinine, Hb, albumin, urea, plasma lipids, glucose, potassium, calcium, and phosphorus in the IDT group (detailed data are provided in Table 7). A total of 11 AEs (adverse events) were recorded in the IDT group during the rehabilitation program, while 15 AEs were recorded in the control group. Given the overall low incidence of adverse events, formal statistical testing was not feasible (Table 8 shows the % incidence of individual AEs).

## 4. Discussion

The primary outcome of this randomized pilot study was to evaluate changes in AS following supervised ambulatory aerobic IDT in HD patients. Results showed that 20 weeks of IDT significantly reduced AS as measured by the CAVI parameter in the exercise group compared with usual-care controls. Several between-group differences reflect a worsening in the control group as much as an improvement in the IDT group. As mentioned in the introduction, there is very little information on the effects of IDT on AS in HD patients. A detailed 2019 meta-analysis [33] that mapped the effectiveness of non-pharmacological interventions on AS in 2166 patients with ESRD reported only four studies that directly addressed the effect of IDT on reducing AS as measured by PWV. Intradialytic exercise compared with no exercise reduced cf-PWV (−1.13 m/s; CI: −2.2 to −0.03; *p* = 0.04). However, recent and larger studies have not confirmed these results, e.g., the randomized controlled trial IHOPE (137 patients) which analyzed in detail the effect of a combination of intra-dialytic protein supplementation and aerobic training on functional fitness and AS as measured by PWV [34]. The PEDAL study (243 HD patients) also found no significant changes in PWV after a 6-month intra-dialytic exercise program [35]. Only the 2021 randomized trial CYCLE-HD (101 HD patients) demonstrated that 6 months of intra-dialytic bicycle training led not only to a significant reduction in left ventricular mass, but also to a significant reduction in PWV in the exercise group [27]. Several recent studies indicate that intradialytic exercise programming results in a clinically meaningful improvement in pulse wave velocity, a component of arterial stiffness. Improvements in several physiological measures of cardiovascular health, including diastolic blood pressure, left ventricular ejection fraction, and heart rate variability (measured by the low-frequency/high-frequency ratio), have also been observed. However, the effects of intradialytic exercise on major adverse cardiovascular events remain uncertain [5]. Some inconsistency in the above results in AS measurement may lie in different measurement techniques, as well as in the duration, type and intensity of exercise, and also in the level of adherence and compliance of patients to the prescribed program. Measurement of AS using PWV is still the gold standard and an independent determinant of functional capacity and exercise intolerance in patients with chronic kidney disease [15]. For example, a 1 m/s increase in PWV increases the overall mortality rate by up to 15% [36]. The present study is the first randomized trial to use CAVI to measure and assess the effects of regular IDT on AS in patients in an outpatient HD program. CAVI was first introduced into clinical practice about two decades ago and has since been widely used in a number of clinical trials, largely focused on measuring AS in patients with pre-existing cardiovascular disease or in subjects at high CV risk [37]. In addition, CAVI has recently been shown to be a valuable predictor of CV morbidity and all-cause mortality [38]. Methodologically, CAVI is based on PWV measurements, but since it is mathematically derived from the stiffness parameter β and the Bramwell–Hill equation, it is completely independent of blood pressure at the time of measurement [28,39]. In addition, it is clear that CAVI reflects the functional stiffness caused by the contraction or relaxation of arterial smooth muscle [39]. Compared to PWV, CAVI therefore provides much more accurate information about arterial stiffness, which should be considered an extremely important fact in view of the complicated cardiovascular condition and characteristic hemodynamic instability in patients with chronic renal insufficiency [40,41]. In patients with end-stage renal disease, increased CAVI has been reported to be a predictor of primary CV events [42] and reflects the progression of renal dysfunction and increased mortality in patients with chronic kidney disease [43]. Takenaka et al. (2008) established a CAVI score ≥7.55 as a cut-off value for suspicion of CV disease in hemodialysis patients [40]. A CAVI value ≥10 signals the risk of future renal dysfunction in asymptomatic individuals [44]. During physical exertion, optimal interaction between the individual compartments of the transport system (i.e., the pulmonary and cardiovascular system, and the periphery) is a basic prerequisite for meeting increased metabolic demands. This implies that functional capacity is closely related to the level of AS in both healthy individuals and patients with CV disease [45,46]. If the vasodilator properties of the arterial system are impaired due to increased AS, this must necessarily be reflected in a deterioration of cardiopulmonary capacity, lower peripheral O_2_ extraction, and exercise intolerance [47,48]. This fact was related to an important secondary objective of this study—the evaluation of the effect of IDT on functional capacity, expressed as peak oxygen uptake (VO_2peak_). The results showed a significant increase in VO_2peak_ and VO_2_VT-1 values, which can be understood as an improvement in physical fitness at the maximal and submaximal level. This finding corresponds to the vast majority of previous clinical studies in which similar IDT programs were used [49,50]. The overall positive effect of IDT on the health status of patients can also be attributed to the lower percentage of adverse events in the exercise group compared to the control group. Reduced VO_2peak_ values are not only a sign of impaired physiological functions, but are closely related also to the length of survival. The limit for an independent lifestyle and a higher survival rate is the value of VO_2peak_ = 17.5 mL/kg/min, i.e., 5 MET [51,52,53,54]. In contrast, an improvement in cardiorespiratory fitness by just 1 metabolic equivalent (i.e., 3.5 mL O_2_/kg/min) corresponds to a 16% reduction in mortality [55]. In addition to parameters of functional capacity or muscle strength in HD patients, monitoring of BP changes is important. After the end of the IDT program, a decrease in SBP and DBP was noted in the exercise group, but a significant decrease was observed only in DBP. In this context, it should be emphasized that the results of BP changes due to regular physical activity in HD patients are not consistent in their conclusions. A meta-analysis of 33 studies in 1254 HD patients found a significant decrease in SBP and DBP due to combined (aerobic + strength) training [50]. Similar results are presented by a meta-analysis (27 randomized studies in 1215 HD patients), i.e., a decrease in SBP and DBP due to aerobic training (18). In contrast, another meta-analysis (78 studies in 3326 patients) reported 22 studies evaluating changes in SBP and DBP due to different types of training: a significant reduction was demonstrated only in DBP due to combined training, while other modalities (aerobic, strength, combined or electromyostimulation) had no effect on SBP (19). This is in accordance with the results of our study. Gait speed is a well-known method to evaluate skeletal muscle function in patients with hemodialysis [51]. A significant increase in the distance covered in the 6 MWT in the IDT group was also observed. Walking ability is a crucial factor in daily activities and self-care. Therefore, a decrease in physical functions, including walking, is a significant independent predictor of CV risk and mortality in HD patients, and an increase in the distance covered is considered an important indicator of improvement in submaximal endurance [22,23,24,56]. A change of 0.1 m/s in gait speed is considered a clinically significant change that is sufficient to improve independent activities of daily living. Furthermore, previous studies have demonstrated that muscle strength is more critical than muscle mass regarding outcomes in patients undergoing hemodialysis [52]. Accelerated dysregulation of the immune system in patients with chronic kidney disease is the cause of activation of a cascade of pro-inflammatory processes and subsequent potentiation of structural-functional pathological changes in the arterial wall [57,58,59]. CRP, an important acute phase reactant, reflects AS in both healthy individuals and patients with end-stage renal disease and HD [58]. There are a number of strategies to reduce inflammatory substances (including CRP) in these patients, e.g., regular exercise [22,60]. This assumption was also fulfilled in this study, where after 20 weeks of IDT plasma CRP levels decreased by approximately 22%. Persistently elevated glucose levels are a significant cause of vascular dysfunction in diabetes mellitus, initiating metabolic alterations such as increased levels of advanced glycation end products (AGEs) and reactive oxygen species (ROS). Advanced glycation end products can decrease the availability of nitric oxide (NO), negatively affecting endothelium-dependent vasodilation in diabetes mellitus. The complications of type 2 diabetes mellitus are further driven by the modulation of endothelial nitric oxide synthase (eNOS) and nuclear factor erythroid 2-related factor 2/heme oxygenase-1 (NrF_2_/HO-1) signaling pathways [61]. Clearance of uremic toxins is a standard indicator of dialysis efficacy. In our study, after 20 weeks of IDT significant improvements were shown in the Kt/V and urea reduction ratio (URR) parameters, which can be considered as an improvement in the overall “wash-out” effect of dialytic procedure and corresponds with current reports [21,22,62]. Acute application of intra-dialytic exercise is cardioprotective, reducing myocardial stunning compared with standard HD. The cardioprotection offered by this intervention is of significant clinical importance, as it may prevent overt cardiac dysfunction and reduce the risk of major cardiac events and mortality associated with the recurrent transient left ventricle dysfunction imposed by repetitive HD [63]. The beneficial effects of IDT on functional capacity in dialysis patients influence also the changes in QoL [21,22,62,64]. The evaluation of the KDQoL-36 questionnaires in our study demonstrated a certain benefit on some aspects of QoL in the IDT group, i.e., social support, general health, and well-being. In particular, the significant improvement in well-being (*p* < 0.001) can reflect an overall stabilization of health status. In contrast, no significant changes in QoL occurred in the control group. There is no doubt that the critical points of effective implementation of IDT are not only determining the safe intensity of the workload, but also the duration of the training program. The aforementioned meta-analysis [22] states that exercise programs for dialysis patients longer than 12 weeks improve functional parameters significantly more than exercises up to 12 weeks. At the same time, it seems that moderate to high intensity training leads to a more significant improvement in functional capacity and distance covered in 6 MWT compared to training of mild or mild-to-moderate intensity [22,65,66]. It should be emphasized that a significant share in the predominantly positive outcomes in the exercise group in our study had a relatively high adherence to the IDT program (avg. 79%), which—as mentioned above—is one of the pillars of the success of exercise-based rehabilitation programs for dialysis patients [24,27]. In contrast, f.e., the large randomized PEDAL study reported very low adherence and compliance (18% and 47%, respectively), which undoubtedly had a negative impact on the results [35]. The amount of verified information and positive experiences about the effectiveness of physical activity in end-stage renal disease or dialysis patients is well-known. However, rehabilitation programs for these patients are still not part of standard (routine) health care [19,67]. The primary obstacle is, of course, a significant level of inactivity and extremely poor motivation of patients, where a significant role has the constant psychological pressure of awareness of the presence of an incurable chronic disease, a number of concomitant comorbidities, and a gradual reduction in self-sufficiency [5]. When we add to this the limited (sometimes zero) interest of the medical community and the often-mentioned high costs of organizing and providing exercise-based renal rehabilitation care, a problem arises that is still very difficult to solve [35,68].

### 4.1. Strengths

To our knowledge, this is the first randomized study that demonstrates the effect of supervised intradialytic aerobic training on AS using the CAVI parameter in HD patients. The most valuable feature of CAVI is that the BP does not affect it at the time of measurement, and this is extremely important, considering the well-known hemodynamic instability in HD patients.

### 4.2. Limitations

We are aware that the results of AS measurement using CAVI are preliminary and should be interpreted with caution. There were several limiting factors, primarily the limited number of patients in both groups. In addition, compared to PWV, there is still insufficient experience with CAVI measurement in HD patients. The average baseline CAVI in the IDT group was 8.57, which according to reference values corresponds to only a moderate degree of CV risk [69], and the resulting significant reduction of 0.45 at the end of the program was not very high (*p* = 0.037) and does not represent a clinically meaningful improvement. It is therefore debatable what would be the rate of change in CAVI in patients with a more severe degree of CV impairment. We acknowledge that with a final sample size of 36 participants, the study is underpowered to detect small-to-moderate effect sizes, increasing the risk of type II errors (false negatives) for nonsignificant findings. Conversely, for the significant differences observed in the primary CAVI outcome, the actual effect size was insufficiently large (Cohen’s *d* = 0.232). Measurement of endothelial function (e.g., flow-mediated dilation), which was not part of the study, would probably bring more objectivity. Finally, it is necessary to take into account the fact that the most extensive study and clinical experience with this parameter comes from data from Asian populations. Therefore, it is necessary to focus further research activities related to CAVI on other ethnic groups.

## 5. Conclusions

Given the proven close connection between pro-inflammatory factors, cardiovascular damage and mortality in HD patients, it is somewhat surprising that so far the measurement of AS has been given rather limited attention. At the same time, the pathological mechanisms and determinants of AS have been largely identified today. Therefore, regular monitoring of AS using CAVI, PWV or other validated methods appears to be a very important element of efforts to prevent CV risks in HD (and end-stage renal disease) patients. This study demonstrated a positive effect of IDT on AS, improvement of important indicators of functional fitness at peak and submaximal levels, increased efficiency of the dialysis procedure, reduction of pro-inflammatory state, and partial improvement of some QoL parameters. These results reflect multiple beneficial effects of exercise, vital for the health status of HD patients. Moreover, the positive findings in exercising patients contrast with the results of the control group, in which there were no dramatic negative changes in the monitored parameters, but at the same time, their condition reflects a persistent increased CV risk. Based on our results, the CAVI parameter can be recommended as a reliable and simple alternative for monitoring AS in HD patients.

## Figures and Tables

**Table 1:** Baseline characteristic of the study population including comorbidities.

Baseline Characteristics	Control *n* = 14	IDT *n* = 22	Total *n* = 36
Age			
Mean (SD)	56.64 (16.11)	53.68 (15.24)	54.83 (15.42)
Gender			
N (%) Female	7 (50.00)	5 (22.73)	12 (33.33)
Weight (kg)			
Mean (SD)	85.89 (22.98)	77.18 (17.03)	80.57 (19.71)
BMI (kg/m^2^)			
Mean (SD)	30.04 (6.64)	26.81 (6.36)	28.07 (6.57)
SBP (mm Hg)			
Mean (SD)	131.50 (22.01)	145.09 (15.72)	139.81 (19.33)
DBP (mm Hg)			
Mean (SD)	79.14 (10.84)	91.64 (15.66)	86.78 (15.13)
HR REST (bpm)			
Mean (SD)	76.71 (7.41)	74.91 (7.35)	75.61 (7.32)
Anemia			
***n*** (%) Yes	10 (71.43)	16 (72.73)	26 (72.22)
Coronary artery disease			
***n*** (%) Yes	3 (21.43)	2 (9.09)	5 (13.89)
Diabetes mellitus type 2			
***n*** (%) Yes	8 (57.14)	4 (18.18)	12 (33.33)
Dyslipidemia			
***n*** (%) Yes	8 (57.14)	7 (31.82)	15 (41.67)
Hyperparathyroidism			
***n*** (%) Yes	7 (50.00)	12 (54.55)	19 (52.78)
Hypertension			
***n*** (%) Yes	11 (78.57)	17 (77.27)	28 (77.78)
Hyperuricemia			
***n*** (%) Yes	6 (42.86)	6 (27.27)	12 (33.33)
Underlying CRI etiology			
Hypertension			
***n*** (%) Yes	9 (64.29)	13 (59.09)	22 (61.11)
Diabetic angiosclerosis			
***n*** (%) Yes	3 (21.43)	4 (18.18)	7 (19.44)
Glomerulonephritis			
***n*** (%) Yes	2 (14.29)	2 (9.09)	4 (11.11)
Chronic pyelonephritis			
***n*** (%) Yes	0 (0.00)	1 (4.55)	1 (2.78)
Unidentified kidney disease			
***n*** (%) Yes	0 (0.00)	2 (9.09)	2 (5.56)

IDT, intradialytic training; BMI, body mass index; DBP, diastolic blood pressure; HR REST, heart rate at rest; SBP, systolic blood pressure; SD, standard deviation.

**Table 2:** Changes in CAVI after 20 weeks from the onset of intervention in the intra-dialytic aerobic training group and control group.

CAVI	Baseline	20 Weeks	Regression Coefficient *	*p* Value
Control	8.31 (1.70)	8.56 (1.84)	−0.65 (−1.26 to −0.04)	0.037
IDT	8.57 (1.37)	8.12 (1.93)		

IDT, intradialytic training; CAVI, cardio-ankle vascular index. * Regression coefficient describing the difference in means (95% confidence interval) between the control group (reference) and IDT group with respect to the adjusting for age, gender, and hypertension.

**Table 3:** Changes in spiroergometry after 20 weeks from the onset of intervention in the intra-dialytic aerobic training group and control group.

Spiroergometry	Baseline	20 Weeks	Regression Coefficient *	*p* Value
HR_peak_				
Control	113.00 (23.92)	111.95 (21.13)	−1.54	0.791
IDT	117.86 (23.20)	116.91 (23.50)	(−13.31 to 10.23)	
W_peak_/kg				
Control	0.98 (0.42)	1.20 (0.49)	0.05	0.353
IDT	0.95 (0.44)	1.25 (0.44)	(−0.06 to 0.15)	
HR at VT-1				
Control	92.50 (16.48)	85.23 (11.55)	−4.66	0.247
IDT	99.57 (20.09)	88.86 (12.62)	(−12.70 to 3.39)	
VO_2_ at VT-1 (mL/kg/min)				
Control	9.14 (3.66)	9.66 (4.26)	1.41	< 0.001
IDT	8.56 (3.45)	10.57 (4.74)	(0.69 to 2.13)	
VO_2peak_/kg (mL/kg/min)				
Control	14.15 (4.72)	15.73 (5.85)	2.04	0.015
IDT	13.03 (5.29)	17.11 (5.43)	(0.42 to 3.67)	

IDT, intradialytic training; HR_peak_, peak heart rate; VO_2_ at VT-1, O_2_ uptake the first ventilatory threshold; VO_2peak_/kg, peak O_2_ per kilogram, ventilatory threshold 1; W_peak_/kg, workload per kilogram. * Regression coefficient describing the difference in means (95% confidence interval) between the control group (reference) and IDT group with respect to the adjusting for age, gender, and hypertension.

**Table 4:** Changes in 6 MWT after 20 weeks from the onset of intervention in the intra-dialytic aerobic training group and control group.

6 MWT	Baseline	20 Weeks	Regression Coefficient *	*p* Value
HR mean (6 min CWT)				
Control	94.40 (14.20)	98.11 (15.42)	2.95	0.532
IDT	92.08 (16.54)	99.55 (17.65)	(−6.56 to 12.45)	
RPE mean (6–20)				
Control	12.90 (2.34)	10.88 (3.50)	1.42	0.096
IDT	11.13 (2.01)	10.46 (2.73)	(−0.26 to 3.10)	
Predicted distance				
Control	630.60 (67.38)	630.49 (69.49)	7.34	0.419
IDT	653.58 (205.07)	658.32 (196.90)	(−10.93 to 25.60)	
Distance walked				
Control	357.50 (94.63)	340.79 (100.04)	66.23	0.005
IDT	331.96 (190.62)	390.67 (237.11)	(21.13 to 111.33)	

IDT, intradialytic training; 6 MWT, 6 min walk test; HR mean, mean heart rate; RPE, ratio of perceived exertion. * Regression coefficient describing the difference in means (95% confidence interval) between the control group (reference) and IDT group with respect to the adjusting for age, gender, and hypertension; Predicted distance, reference value calculated using standardized regression equations; Distance walked, real, measured distance.

**Table 5:** Changes in hemodynamic parameters after 20 weeks from the onset of intervention in the intra-dialytic aerobic training group and control group.

BP, HR	Baseline	20 Weeks	Regression Coefficient *	*p* Value
SBP (mmHg)				
Control	131.50 (22.01)	136.14 (24.37)	−12.99	0.023
IDT	145.09 (15.72)	138.05 (14.54)	(−24.10 to −1.88)	
DBP (mmHg)				
Control	79.14 (10.84)	84.29 (10.89)	−13.40	0.001
IDT	91.64 (15.66)	83.41 (9.31)	(−20.91 to −5.89)	
HR REST (bpm)				
Control	76.71 (7.41)	79.36 (8.64)	−3.40	0.081
IDT	74.91 (7.35)	74.18 (6.38)	(−7.24 to 0.45)	

IDT, intra-dialytic training; BP, blood pressure; DBP, diastolic blood pressure; HR, heart rate; HR REST, heart rate at rest; SBP, systolic blood pressure. * Regression coefficient describing the difference in means (95% confidence interval) between the control group (reference) and IDT group with respect to the adjusting for age, gender, and hypertension.

**Table 6:** Changes in KDQoL-36 after 20 weeks from the onset of intervention in the intra-dialytic aerobic training group and control group.

KDQOL-36	Baseline	20 Weeks	Regression Coefficient *	*p* Value
Symptoms				
Control	81.36 (9.53)	82.10 (7.00)	−0.19	0.959
IDT	77.72 (12.03)	78.61 (12.21)	(−7.70 to 7.33)	
Effects of kidney disease				
Control	52.89 (18.28)	55.34 (18.06)	3.63	0.222
IDT	54.30 (15.30)	60.77 (15.81)	(−2.31 to 9.57)	
Burden of kidney disease				
Control	40.55 (23.48)	42.15 (25.45)	4.00	0.284
IDT	41.62 (23.95)	47.03 (23.13)	(−3.47 to 11.47)	
Work status				
Control	35.71 (41.27)	39.29 (40.09)	−8.44	0.123
IDT	27.27 (40.02)	22.73 (36.93)	(−19.3 to 2.43)	
Cognitive function				
Control	84.05 (14.52)	88.10 (10.35)	3.25	0.478
IDT	81.60 (12.18)	87.08 (10.98)	(−5.97 to 12.46)	
Quality of social interaction				
Control	74.03 (16.89)	79.79 (15.61)	−3.13	0.542
IDT	79.13 (14.92)	80.73 (14.45)	(−13.47 to 7.22)	
Sexual function				
Control	21.19 (33.23)	30.27 (41.48)	−8.61	0.452
IDT	29.97 (37.68)	31.67 (39.44)	(−31.63 to 14.42)	
Sleep				
Control	63.32 (14.53)	64.14 (18.66)	5.46	0.198
IDT	59.80 (15.43)	65.07 (20.35)	(−2.99 to 13.91)	
Social support				
Control	83.52 (14.23)	79.65 (18.55)	11.75	0.022
IDT	76.73 (18.60)	83.98 (17.32)	(1.84 to 21.66)	
Dialysis staff encouragement				
Control	96.43 (9.08)	97.32 (7.24)	2.25	0.678
IDT	84.81 (21.79)	88.64 (16.32)	(−8.72 to 13.23)	
Patient satisfaction				
Control	84.77 (18.93)	88.49 (13.62)	1.21	0.830
IDT	76.97 (17.66)	81.17 (16.23)	(−10.18 to 12.60)	
Physical functioning				
Control	53.86 (26.03)	54.86 (27.58)	2.90	0.530
IDT	51.55 (27.86)	56.41 (32.46)	(−6.40 to 12.20)	
Role—physical				
Control	35.71 (32.10)	39.29 (36.31)	7.47	0.481
IDT	20.45 (23.95)	32.95 (33.08)	(−13.91 to 28.85)	
Pain				
Control	57.46 (25.51)	59.38 (27.16)	3.86	0.559
IDT	61.07 (23.40)	67.24 (25.94)	(−9.45 to 17.16)	
General health				
Control	40.36 (26.20)	38.21 (27.78)	9.10	0.008
IDT	27.27 (14.20)	34.09 (17.02)	(2.50 to 15.70)	
Well being				
Control	77.43 (15.50)	65.71 (17.38)	15.29	<0.001
IDT	70.55 (11.55)	72.73 (12.93)	(7.55 to 23.03)	
Role—emotional				
Control	51.64 (31.80)	71.00 (32.46)	−11.23	0.294
IDT	66.01 (32.32)	79.35 (32.29)	(−32.66 to 10.21)	
Social function				
Control	64.93 (25.30)	66.31 (21.47)	6.11	0.235
IDT	59.74 (22.03)	69.59 (24.36)	(−4.18 to 16.40)	
Energy				
Control	50.00 (17.54)	51.43 (17.59)	2.68	0.353
IDT	48.86 (14.30)	53.12 (15.45)	(−3.12 to 8.48)	

IDT, intra-dialytic training; KDQoL-36, kidney disease quality of life. * Regression coefficient describing the difference in means (95% confidence interval) between the control group (reference) and IDT group with respect to the adjusting for age, gender, and hypertension.

**Table 7:** Changes in laboratory parameters after 20 weeks from the onset of intervention in the intra-dialytic aerobic training group and control group.

Laboratory Parameters	Baseline	20 Weeks	Regression Coefficient *	*p* Value
Hb [g/L]				
Control	116.86 (8.94)	113.43 (9.75)	7.12	0.132
IDT	109.75 (12.75)	113.86 (17.00)	(−2.25 to 16.50)	
Albumin [g/L]				
Control	43.16 (3.89)	44.12 (3.14)	−1.30	0.181
IDT	41.68 (3.53)	41.34 (2.70)	(−3.23 to 0.64)	
Calcium [mmol/L]				
Control	2.22 (0.11)	2.31 (0.14)	−0.07	0.238
IDT	2.27 (0.23)	2.29 (0.22)	(−0.20 to 0.05)	
Phosphorus [mmol/L]				
Control	2.06 (0.32)	1.98 (0.25)	−0.18	0.320
IDT	2.06 (0.54)	1.81 (0.53)	(−0.54 to 0.18)	
Creatinine [μmol/L]				
Control	692.36 (124.91)	694.86 (123.53)	−59.62	0.157
IDT	986.32 (246.74)	947.91 (241.53)	(−143.40 to 24.16)	
CRP [mg/L]				
Control	8.92 (6.11)	9.97 (6.53)	−2.44	0.025
IDT	7.45 (6.15)	5.83 (4.73)	(−4.56 to −0.32)	
Potassium [mmol/L]				
Control	4.03 (0.46)	4.11 (0.51)	−0.17	0.331
IDT	4.97 (0.72)	4.87 (0.65)	(−0.52 to 0.18)	
Total cholesterol				
Control	5.26 (0.80)	5.19 (0.77)	0.13	0.563
IDT	4.01 (1.08)	4.15 (0.97)	(−0.33 to 0.60)	
TAG				
Control	2.34 (0.78)	2.26 (0.76)	−0.15	0.430
IDT	1.67 (1.00)	1.48 (0.87)	(−0.55 to 0.24)	
Glucose				
Control	5.76 (0.99)	5.62 (0.88)	−0.28	0.484
IDT	5.97 (2.89)	5.50 (2.47)	(−1.10 to 0.53)	
Urea [mmol/L]				
Control	26.31 (6.18)	28.09 (4.16)	−3.53	0.089
IDT	25.55 (6.01)	24.18 (5.30)	(−7.63 to 0.57)	
URR				
Control	73.79 (5.67)	72.18 (5.98)	3.78	< 0.001
IDT	74.47 (5.67)	76.28 (5.68)	(2.07 to 5.48)	
Kt/V				
Control	1.45 (0.58)	1.37 (0.53)	0.16	0.001
IDT	1.62 (0.31)	1.71 (0.30)	(0.07 to 0.24)	
Parathyroid hormone (pmol/L)				
Control	41.81 (27.25)	44.46 (26.47)	−3.56	0.538
IDT	43.90 (29.24)	41.91 (27.96)	(−15.21 to 8.1)	

IDT, intra-dialytic training; CRP, C-reactive protein; Hb, hemoglobin; Kt/V, K (dialyzer clearance of urea), t (dialysis time), V (volume of distribution of urea); TAG, triacylglycerols; URR, urea reduction ratio. * Regression coefficient describing the difference in means (95% confidence interval) between the control group (reference) and IDT group with respect to the adjusting for age, gender, and hypertension.

**Table 8:** Adverse events in the intra-dialytic aerobic training group and control group after 20 weeks from the onset of intervention *n* (%) (Yes).

Adverse Events *n* (%) (Yes)	Control *n* = 14	IDT *n* = 22	Both Groups *n* = 36
Hypotension	2 (14.29)	0 (0.00)	2 (5.56)
Muscle cramps	2 (14.29)	2 (9.09)	4 (11.11)
Low-back pain	4 (28.57)	3 (13.64)	7 (19.44)
Musculo-skeletal discomfort	3 (21.43)	2 (9.09)	5 (13.89)
Abdominal pain (unspecified)	3 (21.43)	1 (4.55)	4 (11.11)
Dyspnea	0 (0.00)	1 (4.55)	1 (2.78)
Chest pain	0 (0.00)	0 (0.00)	0 (0.00)
Upper respiratory tract infection	1 (7.14)	2 (9.09)	3 (8.33)
Total number of adverse events	15	11	26

## Data Availability

The dataset can be made available from the corresponding author by request.
